# Sodium intake in US ethnic subgroups and potential impact of a new sodium reduction technology: NHANES Dietary Modeling

**DOI:** 10.1186/1475-2891-13-120

**Published:** 2014-12-18

**Authors:** Victor L Fulgoni, Sanjiv Agarwal, Lisa Spence, Priscilla Samuel

**Affiliations:** Nutrition Impact LLC, Battle Creek, MI USA; NutriScience LLC, 901 Heatherwood Drive, East Norriton, PA USA; Tate & Lyle Ingredients Americas LLC, Decatur, IL USA

**Keywords:** Sodium intake, Ethnic subgroups, Sodium reduction modeling, NHANES, Sodium reduction technology

## Abstract

**Background:**

Because excessive dietary sodium intake is a major contributor to hypertension, a reduction in dietary sodium has been recommended for the US population. Using the National Health and Nutrition Examination Survey (NHANES) 2007–2010 data, we estimated current sodium intake in US population ethnic subgroups and modeled the potential impact of a new sodium reduction technology on sodium intake.

**Methods:**

NHANES 2007–2010 data were analyzed using The National Cancer Institute method to estimate usual intake in population subgroups. Potential impact of SODA-LO® Salt Microspheres sodium reduction technology on sodium intake was modeled using suggested sodium reductions of 20-30% in 953 foods and assuming various market penetrations. SAS 9.2, SUDAAN 11, and NHANES survey weights were used in all calculations with assessment across age, gender and ethnic groups.

**Results:**

Current sodium intake across all population subgroups exceeds the Dietary Guidelines 2010 recommendations and has not changed during the last decade. However, sodium intake measured as a function of food intake has decreased significantly during the last decade for all ethnicities. “Grain Products” and “Meat, Poultry, Fish, & Mixtures” contribute about 2/3^rd^ of total sodium intake. Sodium reduction, using SODA-LO® Salt Microspheres sodium reduction technology (with 100% market penetration) was estimated to be 185–323 mg/day or 6.3-8.4% of intake depending upon age, gender and ethnic group.

**Conclusions:**

Current sodium intake in US ethnic subgroups exceeds the recommendations and sodium reduction technologies could potentially help reduce dietary sodium intake among those groups.

## Background

The prevalence of hypertension in America has increased over the past 20 years in men, women, Blacks, and Whites [1]. Based on 2007 to 2010 data, 33% (about 78 million) of US adults have hypertension and African American adults have among the highest prevalence of hypertension (44%) in the world [2]. In 2010, high blood pressure was estimated to be responsible for $156 billion in direct and indirect health care cost [3]. Because excessive dietary sodium intake is a significant contributor to hypertension [3–9], limiting sodium intake has been recommended for the US population by US public health agencies and other expert scientific organizations, such as the American Heart Association [5, 7, 10, 11]. The Dietary Guidelines for Americans 2010 [11] recommend a maximum dietary sodium intake of 2,300 mg/day for the general population and 1500 mg/day for at-risk groups, including African Americans, older adults (age 51 years and above), and persons of any age with hypertension, diabetes, or chronic kidney disease (about half of the US population). The World Health Organization (WHO) [12] recommends adults consume less than 2,000 mg of sodium, or 5 grams of salt. Regardless of these recommendations, dietary sodium intake in the US is well above that needed for physiological function and is greater than recommended.

Sodium is primarily consumed as sodium chloride and the majority of sodium in the diet comes from sodium added during food processing as an ingredient for flavor, processing aid, and for food safety purposes [11, 13]. Processed foods contribute more than 75% of dietary sodium intake in the US diet; about 10% of dietary sodium occurs naturally in foods and another 5-10% is discretionary salt [4].

In this study, we used the most recent (2007–2010) data from the National Health and Nutrition Examination Survey (NHANES) to estimate the current sodium intake in population subgroups and modeled the potential impact of SODA-LO® Salt Microspheres sodium reduction technology on sodium intake. SODA-LO® is a sodium-reduction ingredient that can reduce sodium in certain applications through its technology that turns standard salt crystals into free-flowing, hollow salt microspheres, which efficiently delivers salt taste and functionality by maximizing surface area.

## Methods

### Study population

NHANES, a large dietary survey of a nationally representative sample of the non-institutionalized US population, was used to assess sodium intake and its sources in the diet of ethnic subgroups in the US population [14]. The NHANES data are collected and released by the National Center for Health Statistics (NCHS) of the Center for Disease Control and Prevention (CDC), every two years. All participants or proxies provided written informed consent and the Research Ethics Review Board at the NCHS approved the survey protocol. Dietary intake data with reliable 24-hour recall dietary interviews (day one via in-person interview at the Mobile Examination Center and day two via telephone interview) using USDA’s automated multiple-pass method (AMPM) were used. The data from NHANES 2007–2008 and 2009–2010 were combined for the analyses [15]. The combined sample included 3,626 Mexican American; 5,559 other Hispanic; 7,369 non-Hispanic White and 3,568 non-Hispanic Black participants ages 2 years and older. Children under age 2 years and pregnant and/or lactating females were excluded from the analyses.

### Estimation of sodium intake

The USDA Nutrient Database for Standard Reference (SR) Releases 22 & 24 were used in conjunction with the Food & Nutrient Database for Dietary Studies (FNDDS) versions 4.1 & 5.0, to determine the sodium derived from foods consumed by NHANES 2007–2008 and NHANES 2009–2010 participants respectively [16–19]. Unadjusted sodium values were used in all analyses. The mean usual intakes (long-run average daily intakes) of sodium from all foods were determined using the National Cancer Institute (NCI) method [20] for a single dietary component, because sodium is consumed at some level on most days. All analyses were adjusted for the complex survey design of NHANES using the appropriate sample weight. Covariates in the usual intake models included age and gender groups, day of the week of dietary recall (weekend/weekday), and interview sequence of the dietary recall (in person versus via telephone).

### Estimation of food sources of sodium

Food groups for NHANES 2007–2008 and NHANES 2009–2010 dietary intake data were defined using the USDA FNDDS 4.1 & FNDDS 5.0 databases, respectively [16, 18]. Data for over 7000 foods were collapsed into 9 broad categories of FNDDS food groups. Sodium consumption (mg/day) and amount of sodium as percent of total dietary intake (mg/kcal and mg/g food) were computed for all FNDDS food groups.

### Sodium intake modeling analysis

SODA-LO® Salt Microspheres is a sodium-reduction ingredient which can reduce sodium in certain applications through its technology that turns standard salt crystals into free-flowing, hollow salt microspheres which efficiently delivers salt taste and functionality by maximizing surface area. A 20% to 30% reduction in sodium content in 953 foods (17 foods in “Milk & Milk Products” for 20% reduction, 304 foods in “Meat, Poultry, Fish & Mixtures” for 20 to 25% reduction, 20 foods in “Egg” for 25% reduction, 30 foods in “Dry Beans, Peas, Other Legumes, Nuts & Seeds” for 25% reduction, 511 foods in “Grain Products” for 25% reduction, 35 foods in “Vegetable” for 20 to 30% reduction, and 36 foods in “Fats, Oils & Salad Dressings” for 25% reduction) was modeled. Various scenarios for potential reduction in usual intake of sodium were then computed by using a 0-100% reduction factor and 10-100% market penetration. The individual reductions were computed for foods using the reduction factor and market penetration factor, and were used to model usual intake after sodium reduction.

### Statistical methods

SAS 9.2 (SASs Institute, Inc.; Cary, NC) and SUDAAN 11 (Research Triangle Institute, Research Triangle Park, NC, USA,) were used for all calculations. NHANES survey weights, strata and primary sampling units were used in all calculations to adjust for oversampling of certain groups, non-response by some selected sample persons, and to adjust for the complex sample design of NHANES to ensure nationally representative results. Data are presented as means ± standard errors (SE). P < 0.01 was considered statistically significant.

## Results

**Figure 1:**
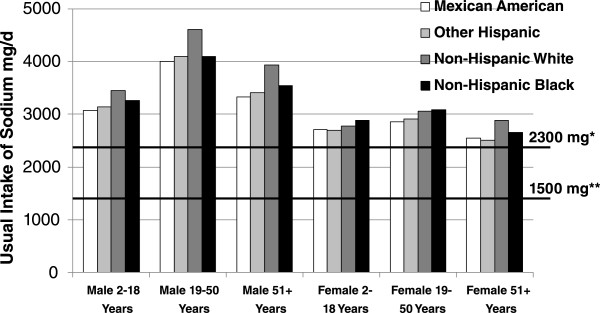
**Usual intake of sodium by age and gender groups in population subgroups.** Data from NHANES 2007–2010. Usual intakes from foods were estimated by using the National Cancer Institute method. *Dietary Guidelines for Americans 2010 recommended level for general population; **Dietary Guidelines for Americans 2010 recommended level for at risk group.

Usual intakes of sodium across age, gender and ethnic groups are shown in Figure 1. Intake of sodium was dependent on age, gender and ethnicity. The usual intake of adults (age 19–50 years) of any gender and ethnicity was higher compared to children (age 2–18 years) and older adults (age 51 years and above) of the same gender and ethnicity. The age related differences in usual intakes were much more pronounced in males than in females of any ethnicity. Males of any age and ethnic group consumed more sodium than females of the corresponding age and ethnic group. Non-Hispanic White (especially males) consumed more sodium than other ethnic groups (Figure 1). Usual intakes of all age, gender and ethnic groups were higher than 2300 mg/day. Intakes of sodium were below 1500 mg/day for less than 5% population of any age, gender and ethnicity (except for older adult Other Hispanic females).

**Figure 2:**
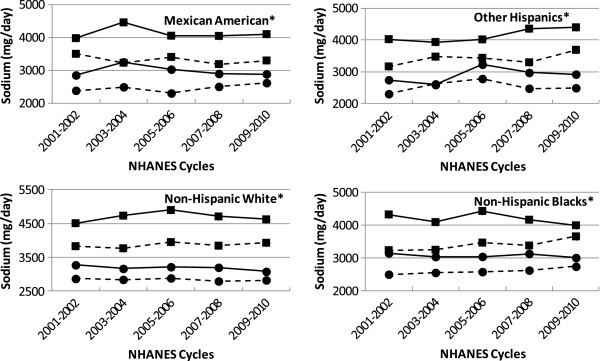
**Sodium intake trends by age and gender groups over 5 NHANES cycles in population subgroups.** Data from NHANES 2001–2010. Square: male, Circles: females, solid line: 19–50 years, dotted line: 51 + years. Usual intakes from foods were estimated by using the National Cancer Institute method. *No change over 5 NHANES cycles, P > 0.01.

Trends in sodium intake over the last 10 years (5 NHANES cycles) among adults (ages 19–50 years) and older adults (age 51 years and above) of different ethnicity are shown in Figure 2. Average sodium intake in each NHANES cycle was higher than 1500 mg/day as well as 2300 mg/day for all adults and older adults irrespective of gender and ethnicity. Moreover, in each NHANES cycle, male adults (ages 19–50 years) and male older adults (age 51 years and above) consistently consumed more sodium than female adults and female older adults respectively in all ethnic groups. Average intake of sodium (mg/day) during the past 5 NHANES cycles did not change significantly (P > 0.01) for adults and older adults for any gender or ethnic group (Figure 2). Similarly, the average intake of sodium among children (ages 2–18 years) over the past 5 NHANES cycles was always higher than 2300 mg/day and did not change significantly (P > 0.01) for any gender and ethnic group (data not presented).

Sodium intake was also measured as a function of energy intake (mg/kcal) and as a function of total food intake (mg/g food) in addition to absolute intake (mg/day) for all NHANES cycles in males and females of all ethnicities. Table 1 shows trends in sodium intake by different measures over the last 10 years (5 NHANES cycles). While there was no change in sodium intake measured as mg/day (absolute amount) and as mg/kcal (function of energy intake) for male or female adults or older adults of any ethnic subgroup, sodium intake measured as a function of food intake (mg/g food) decreased significantly (P < 0.01) among all adult and older adult males and females of all ethnicity (except for older adult male other Hispanic). The sodium intake values in adults (age 19 years and older) of all ethnicity were 3586 mg or 1.68 mg/kcal or 1.56 mg/g food in 2001–2002 and 3607 mg or 1.72 mg/kcal or 1.12 mg/g food in 2009–2010.

**Table 1:** **Sodium intake trends by age and gender groups in population subgroups over 5 NHANES cycles**

Ethnicity	Age (Years)	Gender	Sodium intake trend					
			mg/day		mg/g food		mg/kcal	
			beta*	P**	beta*	P**	beta*	P**
Mexican American	19-50	Male	−19.33	0.7652	−0.10	<0.0001	0.03	0.0342
		Female	−34.38	0.2673	−0.14	<0.0001	0.02	0.0544
	51+	Male	−39.13	0.4792	−0.09	<0.0001	−0.01	0.5585
		Female	53.75	0.1116	−0.13	<0.0001	0.02	0.1729
Other Hispanic	19-50	Male	110.83	0.0913	−0.10	<0.0001	0.04	0.0327
		Female	63.23	0.1697	−0.12	0.0004	0.04	0.0635
	51+	Male	91.07	0.4067	−0.11	0.0508	0.03	0.4286
		Female	26.09	0.6285	−0.14	<0.0001	0.03	0.1452
Non-Hispanic White	19-50	Male	23.42	0.5281	−0.12	<0.0001	0.03	0.0012
		Female	−34.81	0.1798	−0.15	<0.0001	0.01	0.2341
	51+	Male	29.05	0.3438	−0.11	<0.0001	0.00	0.6550
		Female	−13.25	0.4582	−0.13	<0.0001	−0.01	0.1134
Non-Hispanic Black	19-50	Male	−59.10	0.2367	−0.14	<0.0001	0.02	0.1816
		Female	−18.59	0.6458	−0.19	<0.0001	0.02	0.0936
	51+	Male	100.06	0.0619	−0.12	<0.0001	−0.02	0.3977
		Female	56.50	0.1167	−0.14	<0.0001	0.03	0.0242

Data from NHANES 2001–2010. Sodium intake was measured as absolute intake (mg/day) as a function of energy intake (mg/kcal) and as a function of food intake (mg/g food).

*beta – regression coefficient; **P < 0.01 significant.

**Figure 3:**
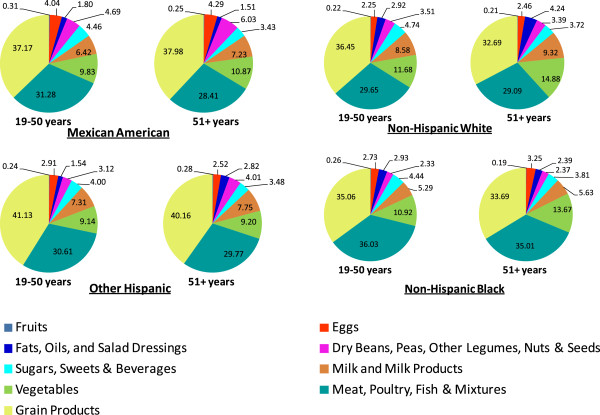
**Dietary sodium contribution from nine FNDDS food groups by age groups in population subgroups.** Data from NHANES 2007–2010. Data is presented as % of total dietary sodium.

The contribution of various food groups (FNDDS defined 9 food groups) to the sodium in the diets of US adults and older adults by population subgroups is shown in Figure 3. No major overall age, gender or ethnicity related differences were noted. “Grain Products” were the top most contributors of dietary sodium, followed by “Meat, Poultry, Fish & Mixtures”. These two food groups contributed 60-70% of total sodium intake in adults and older adults. “Milk & Milk Products”, and “Vegetables”, were the next two major sodium contributors, providing more than 15% of total sodium. These four food groups (“Grain Products”, “Meat, Poultry, Fish & Mixtures”, “Milk & Milk Products”, and “Vegetables”) combined were responsible for more than 85% of total dietary sodium for all ethnic subgroups. The remaining five food groups (“Eggs”, “Dry Beans, Peas, Other Legumes, Nuts & Seeds”, “Fruits”, “Fats, Oils & Salad Dressings”, and “Sugars, Sweets & Beverages”) contributed less than 15% of the sodium in the diet (Figure 3).

Table 2 shows the maximum achievable reduction (using the maximum reduction factor and 100% market penetration) in sodium intake across all food categories to be 185–323 mg (6.3-8.4%). A lower reduction factor and/or lower market penetration would provide lower reductions. A somewhat higher reduction is expected for non-Hispanic Whites and non-Hispanic Blacks compared to Mexican Americans and Other Hispanics (Table 2). “Grain Products” and “Meat, Poultry, Fish & Mixtures” were the main contributors of sodium reduction, contributing to more than 80% of total sodium reduction. Sodium reduction in “Grain Products” contributed to 60-70% of total sodium reduction for Mexican Americans, other Hispanics and non-Hispanic Whites, and 50-60% of total sodium reduction for non-Hispanic Black adults and older adults. Sodium reduction in “Meat, Poultry, Fish & Mixtures” contributed about 16-24% of total sodium reduction for Mexican Americans, other Hispanics and non-Hispanic Whites, and about 25-30% of total sodium reduction for non-Hispanic Blacks adults and older adults (Table 3).

**Table 2:** **Potential sodium intake reduction with SODA-LO® Salt Microspheres (Sodium Replacement Technology) in population subgroups**

Ethnicity	Age	Potential reduction* (mg)	Current intake (mg)	Potential intake after reduction (mg)	% Reduction*
Mexican American	19-50 years	250 ± 14	3558 ± 68	3309 ± 59	6.8 ± 0.3
	51+ years	196 ± 12	2898 ± 86	2702 ± 78	6.8 ± 0.4
Other Hispanic	19-50 years	252 ± 11	3599 ± 45	3347 ± 39	6.9 ± 0.3
	51+ years	185 ± 11	2916 ± 68	2731 ± 63	6.3 ± 0.3
Non-Hispanic White	19-50 years	323 ± 8	3903 ± 55	3581 ± 53	8.3 ± 0.2
	51+ years	242 ± 5	3320 ± 49	3078 ± 47	7.3 ± 0.2
Non-Hispanic Black	19-50 years	296 ± 10	3544 ± 66	3248 ± 60	8.4 ± 0.2
	51+ years	222 ± 9	3046 ± 74	2824 ± 67	7.2 ± 0.2

Data from NHANES 2007–2010. Potential reduction was modeled using 20% to 30% targeted maximum reduction in sodium content in 953 foods with 100% market penetration.

*Average of reductions in individuals.

**Table 3:** **Potential sodium intake reduction with SODA-LO® salt microspheres in population subgroups by FNDDS food groups**

	Potential sodium intake reduction, mg & (%)			
	Mean ± SE			
	Mexican American	Other Hispanic	Non-Hispanic white	Non-Hispanic black
**All foods** (953 foods; 20-30% targeted reduction in sodium content)				
Male, 19–50 Years	294 ± 20 (6.9 ± 0.4)	297 ± 15 (7.0 ± 0.3)	398 ± 13 (8.7 ± 0.2)	338 ± 13 (8.3 ± 0.3)
Male, 51+ Years	236 ± 19 (7.3 ± 0.5)	222 ± 18 (6.7 ± 0.5)	290 ± 7 (7.4 ± 0.2)	258 ± 14 (7.2 ± 0.2)
Female, 19–50 Years	193 ± 10 (6.7 ± 0.3)	198 ± 9 (6.8 ± 0.3)	248 ± 7 (8.0 ± 0.2)	259 ± 14 (8.4 ± 0.3)
Female 51+ Years	161 ± 7 (6.3 ± 0.3)	152 ± 7 (6.0 ± 0.3)	200 ± 6 (7.2 ± 0.2)	195 ± 12 (7.2 ± 0.3)
**Milk & milk products** (17 foods; 20% targeted reduction in sodium content)				
Male, 19–50 Years	2 ± 1 (0.2 ± 0.1)	2 ± 1 (0.2 ± 0.1)	4 ± 1 (0.3 ± 0.1)	0.3 ± 0.2 (0.1 ± 0.0)
Male, 51+ Years	0.2 ± 0.1 (0.02 ± 0.01)	0.3 ± 0.2 (0.1 ± 0.0)	5 ± 1 (0.5 ± 0.1)	1 ± 0 (0.2 ± 0.2)
Female, 19–50 Years	2 ± 1 (0.4 ± 0.2)	2 ± 1 (0.3 ± 0.1)	5 ± 1 (0.5 ± 0.1)	2 ± 1 (0.4 ± 0.1)
Female 51+ Years	5 ± 2 (0.6 ± 0.2)	3 ± 1 (0.4 ± 0.1)	6 ± 1 (0.9 ± 0.1)	2 ± 1 (0.4 ± 0.2)
**Meat, poultry, fish & mixtures** (304 foods; 20-25% targeted reduction in sodium content)				
Male, 19–50 Years	67 ± 9 (4.7 ± 0.5)	72 ± 9 (4.8 ± 0.4)	87 ± 5 (5.5 ± 0.3)	117 ± 7 (7.4 ± 0.3)
Male, 51+ Years	44 ± 6 (4.4 ± 0.6)	39 ± 6 (3.9 ± 0.5)	59 ± 4 (4.5 ± 0.2)	79 ± 5 (5.5 ± 0.3)
Female, 19–50 Years	44 ± 7 (5.1 ± 0.6)	43 ± 5 (4.9 ± 0.5)	53 ± 4 (5.8 ± 0.4)	92 ± 7 (8.2 ± 0.5)
Female 51+ Years	37 ± 6 (4.8 ± 0.5)	31 ± 5 (3.9 ± 0.5)	31 ± 3 (3.9 ± 0.3)	49 ± 5 (5.0 ± 0.5)
**Eggs** (20 foods; 25% targeted reduction in sodium content)				
Male, 19–50 Years	4 ± 2 (1.2 ± 0.5)	3 ± 1 (1.0 ± 0.4)	4 ± 1 (1.6 ± 0.4)	4 ± 2 (1.2 ± 0.5)
Male, 51+ Years	2 ± 1 (0.4 ± 0.3)	1 ± 1 (0.3 ± 0.3)	5 ± 1 (2.0 ± 0.5)	4 ± 1 (1.0 ± 0.4)
Female, 19–50 Years	3 ± 1 (1.3 ± 0.5)	3 ± 1 (1.3 ± 0.4)	3 ± 1 (2.1 ± 0.7)	4 ± 2 (2.0 ± 0.7)
Female 51+ Years	2 ± 1 (0.6 ± 0.3)	1 ± 1 (0.4 ± 0.2)	2 ± 1 (0.7 ± 0.3)	3 ± 2 (0.7 ± 0.4)
**Dry beans, peas, other legumes, nuts & seeds** (30 foods; 25% targeted reduction in sodium content)				
Male, 19–50 Years	5 ± 1 (3.3 ± 0.6)	4 ± 1 (3.0 ± 0.5)	7 ± 1 (9.6 ± 0.8)	3 ± 0.7 (8.4 ± 0.9)
Male, 51+ Years	4 ± 1 (4.3 ± 0.9)	3 ± 1 (4.1 ± 0.8)	8 ± 1 (10.7 ± 0.8)	4 ± 1 (6.6 ± 1.0)
Female, 19–50 Years	2 ± 1 (4.0 ± 0.7)	2 ± 0.4 (4.6 ± 0.6)	5 ± 1 (8.0 ± 0.7)	2 ± 0.5 (7.4 ± 1.3)
Female 51+ Years	2 ± 1 (4.1 ± 0.9)	3 ± 1 (5.1 ± 0.8)	5 ± 0.4 (11.1 ± 0.8)	2 ± 0.4 (7.3 ± 1.0)
**Grain products** (511 foods; 25% targeted reduction in sodium content)				
Male, 19–50 Years	195 ± 16 (13.4 ± 0.8)	195 ± 13 (12.6 ± 0.6)	253 ± 11 (15.1 ± 0.4)	179 ± 10 (13.0 ± 0.4)
Male, 51+ Years	166 ± 13 (14.7 ± 0.6)	158 ± 13 (13.4 ± 0.6)	176 ± 6 (14.4 ± 0.4)	145 ± 12 (13.4 ± 0.5)
Female, 19–50 Years	125 ± 5 (11.2 ± 0.3)	132 ± 6 (11.3 ± 0.4)	156 ± 5 (14.0 ± 0.2)	126 ± 8 (11.5 ± 0.5)
Female 51+ Years	99 ± 6 (11.6 ± 0.6)	100 ± 4 (11.4 ± 0.5)	132 ± 5 (14.1 ± 0.3)	117 ± 7 (13.3 ± 0.5)
**Vegetables** (35 foods; 20-30% targeted reduction in sodium content)				
Male, 19–50 Years	18 ± 2 (5.0 ± 0.4)	17 ± 2 (5.0 ± 0.4)	37 ± 3 (7.1 ± 0.4)	29 ± 3 (8.5 ± 0.7)
Male, 51+ Years	15 ± 2 (4.7 ± 0.6)	14 ± 2 (4.6 ± 0.4)	25 ± 2 (4.7 ± 0.3)	18 ± 2 (4.3 ± 0.4)
Female, 19–50 Years	15 ± 1 (4.4 ± 0.4)	14 ± 1 (5.0 ± 0.4)	20 ± 1 (5.8 ± 0.4)	28 ± 3 (7.8 ± 0.6)
Female 51+ Years	12 ± 2 (3.6 ± 0.7)	10 ± 2 (3.3 ± 0.5)	16 ± 1 (3.9 ± 0.3)	17 ± 2 (5.3 ± 0.6)
**Fats, oils & salad dressings** (36 foods; 25% targeted reduction in sodium content)				
Male, 19–50 Years	4 ± 1 (16.1 ± 1.3)	4 ± 0 (16.4 ± 1.0)	7 ± 1 (14.4 ± 0.9)	5 ± 1 (14.4 ± 0.9)
Male, 51+ Years	4 ± 1 (6.0 ± 1.6)	6 ± 1 (15.8 ± 1.3)	12 ± 1 (15.5 ± 0.6)	8 ± 1 (18.6 ± 0.9)
Female, 19–50 Years	2 ± 0 (11.5 ± 1.0)	3 ± 0 (12.0 ± 0.8)	6 ± 1 (13.5 ± 0.7)	4 ± 1 (13.4 ± 1.2)
Female 51+ Years	4 ± 1 (16.1 ± 0.8)	4 ± 1 (15.6 ± 0.8)	9 ± 1 (13.6 ± 0.6)	6 ± 1(16.4 ± 0.8)

Data from NHANES 2007–2010. Potential reduction was modeled using 20% to 30% targeted maximum reduction in sodium content in 953 foods with 100% market penetration.

## Discussion

Average sodium intake across all age, gender and ethnic subgroups in 2007–2010 significantly exceeded most dietary recommendations, including those of the Dietary Guidelines for Americans 2010, American Heart Association 2010, World Health Organization 2012, Institute of Medicine’s 2005 defined Adequate Intake (AI) or Tolerable Upper Intake Level (UL) and other scientific and public health organizations [4, 11, 12, 21, 22]. The Dietary Guidelines for Americans 2010 recommend that at risk population groups, such as older adults (age 51+ years) and African Americans of any age, should limit sodium intake to 1500 mg/day [11]. However, the average intake of non-Hispanic Blacks of any age/gender (except for female children and older adults) and male older adults of any ethnicity were more than twice the US Dietary Guideline recommendations and only a small proportion of the population (less than 5%) met these recommendations. Sodium intake above recommendations is a global issue as demonstrated by high average sodium intakes in other countries [23]. Current evidence suggests that excessive sodium intake is a risk factor for hypertension and consequent health outcomes, including coronary heart disease (CHD), stroke, and mortality [5–9].

The average intake of sodium was age and gender dependent, with males consuming more sodium than females, and adults (ages 19–50 years) consuming more sodium than children (ages 2–18 years) and older adults (age 51 years and above). This observation is most likely due to higher food and caloric intake among males and adults ages 19–50 years. In addition to age and gender dependence, the average intake of sodium was also dependent on ethnicity. The average intake of sodium was highest for Non-Hispanic Whites followed by non-Hispanic Blacks, and other Hispanics and Mexican Americans in every age and gender group (except for non-Hispanic Black female children and female adults). Earlier studies comparing ethnic subgroups also reported a higher mean intake of sodium among non-Hispanic Whites compared to non-Hispanic Blacks and Mexican Americans [13, 24–26]. These observed differences between ethnic population subgroups may be related to differences in dietary patterns. While non-Hispanic Blacks consume slightly less sodium than non-Hispanic Whites, it is recommended that they limit sodium intake to 1500 mg/day due to their likely sensitivity to sodium with a greater response to blood pressure-raising effects of sodium [27, 11].

Our data on ethnic subgroups also showed that sodium intake has not changed in the past decade and has consistently remained higher than the Dietary Guidelines recommendations. There is very limited data available on sodium intake trends among ethnic subgroups in the US population. An analysis of 38 studies from 1957–2003 did not find any significant temporal trend in 24 h sodium excretion among males or females, or Blacks or Whites participants [28]. Another recent analysis of NHANES data reported a slight decline in mean sodium intake but no change in sodium density during 2003 to 2010 and suggested this to be related to declines in calorie consumption [29]. In the present study, sodium intake was also measured as a function of total food intake and total calorie intake, in addition to absolute intake. While there was no change in the absolute amount of sodium intake, we noted a significant decrease in sodium intake as a function of food intake in all ethnic subgroups over the last decade. This suggests that a reduction in the sodium content of foods occurred, but with a consequent increase in food intake during 2001–2010, potentially offsetting the decrease in sodium and resulting in no change in total sodium intake (mg/day) of the population. This finding emphasizes the need for innovative food technologies to help further reduce the sodium content of foods.

Different foods contain different amounts of sodium, which is either added (for flavor, food processing, and/or food safety) or occurs naturally [4, 11, 13]. However, both sodium content of foods and the frequency of food consumption contribute to sodium intake. Often the problem of excess sodium intake is due to frequently consumed foods which might be moderate in their sodium content [4]. Studies from CDC and NCI [30, 31] analyzing NHANES 2005–2006 data, reported that grain and meat products were the top contributors of sodium in the US diet. The present study, using the most recent NHANES dataset (2007–2010) for various ethnic subgroups, shows that two food groups “Grain Products” and “Meat, Poultry, Fish & Mixtures” jointly provide 2/3 of dietary sodium, and four food groups “Grain Products”, “Meat, Poultry, Fish & Mixtures”, “Milk & Milk Products”, and “Vegetables”, jointly provide 4/5 of the dietary sodium for all age, gender and ethnic subgroups. These data suggest that sodium reduction strategies aiming at “Grain Products” and “Meat, Poultry, Fish & Mixtures”, the two major food group sources of sodium in the diet, could have a bigger impact on dietary sodium reduction compared to other food groups.

Continued high sodium intake across the population, despite consistent recommendations to limit sodium and the accumulating evidence linking excessive sodium intake to hypertension, has led to calls for population wide interventions to reduce sodium in the US diet [13, 32]. However, current sodium intake status (2007–2010 NHANES) and the sodium intake trend (2001–2010 NHANES) data presented above for population subgroups indicate that there has been no significant progress in the reduction of sodium intake. Our present dietary sodium modeling data using SODA-LO® Salt Microspheres, a sodium-reduction ingredient at its potential usage level in 953 foods shows a 185–323 mg/day reduction in sodium intake which translates to about 6.3-8.4% reduction of current sodium intake in the US population by ethnic subgroups. Utilizing this technology could add to the stepwise reduction in sodium content of foods that the food industry has been implementing.

Dietary sodium reduction is an important target for public health improvement, as reduced sodium intake has been demonstrated to reduce blood pressure and is also associated with a reduced risk of stroke and fatal coronary heart disease in adults [5–9, 33, 34]. Dietary sodium reduction is estimated (using statistical modeling) to be cost effective, may potentially improve overall health and provide substantial healthcare cost benefits [35–41]. Using the CHD Policy Model (a computer simulation of heart disease in US adults) to quantify the benefits of sodium reduction, Bibbins-Domingo et al. recently estimated that reducing dietary salt by 3 g (1200 mg sodium) per day would reduce CHD, stroke and myocardial infarction, prevent deaths, and save $10-24 billion in health care costs annually [35]. Interpolation of these data [35] suggest a potential for 0.45 to 0.88 mm Hg reduction in systolic blood pressure and $3.0 to 5.3 billion in reductions in health care cost with a 300 mg/day decrease in sodium intake. The present study demonstrated a possible 185–323 mg/day reduction in current sodium intake using SODA-LO®, which could lead to the 300 mg/day decrease in sodium that is attributed to blood pressure and health care cost reductions. Substantially higher potential health benefits due to sodium reduction are expected in at-risk groups, including African Americans, older adults (age 51 years and above), and persons of any age with hypertension, diabetes, or chronic kidney disease, as they may be more responsive to blood pressure-raising effects of sodium [11].

SODA-LO® Salt Microspheres is a sodium-reduction ingredient that converts standard salt crystals into free-flowing, hollow salt microspheres which efficiently delivers salt taste and functionality by maximizing surface area. As with many other sodium reduction approaches, such a technology could modestly increase the cost of some foods, however the potential health benefits, health improvement and reduced healthcare costs from dietary sodium reduction is expected to vastly out-weigh the cost of the technology. Additionally, use of sodium reduction technologies which do not alter flavor may potentially delay the consumer’s palate adaptation for less salt. However, changing consumer behavior is difficult and some attempts to lower dietary salt intake on an individual basis have largely proved to be ineffective [42]. Moreover, changing the palate may require a significant amount of time; in the interim technologies like SODA-LO® Salt Microspheres can provide an immediate solution for sodium intake reduction. Further research is needed to evaluate the effects of sodium reduction in the marketplace as the sodium reduction technologies, such as SODA-LO®, are introduced.

A major strength of our study is the use of a large nationally representative population sample to assess the total usual intake of sodium with the NCI method. One of the limitations of our study was the cross-sectional nature of NHANES data, which prevents definitive conclusions or causality.

## Conclusions

In conclusion, this study reports that current sodium intake in the US in all ethnic subgroups exceeds public health recommendations. Sodium reduction using the technology of SODA-LO® Salt Microspheres could potentially reduce dietary sodium intake by 6-8% in these subgroups.

## Abbreviations

AI: Adequate intake
AMPM: Automated multiple-pass method
CDC: Center for disease control and prevention
CHD: Coronary heart disease
FNDDS: Food & nutrient database for dietary studies
NCHS: National center for health statistics
NCI: National Cancer Institute
NHANES: National Health and Nutrition Examination Survey
SE: Standard error
SR: Standard release
UL: Tolerable upper intake level
WHO: World Health Organization.
